# CTLA4 is expressed on mature dendritic cells derived from human monocytes and influences their maturation and antigen presentation

**DOI:** 10.1186/1471-2172-12-21

**Published:** 2011-03-18

**Authors:** Xiong B Wang, Zhong Z Fan, Doina Anton, Annika V Vollenhoven, Zhen H Ni, Xiao F Chen, Ann K Lefvert

**Affiliations:** 1 Laboratory center, Putuo Hospital, Shanghai University of Traditional Chinese Medicine. 200062, China; 2 Department of Thoracic Surgery, Shanghai Pulmonary Hospital, Tongji University, China; 3 Center of Molecular Medicine, Karolinska Institute, SE-17176, Sweden

## Abstract

**Background:**

Dendritic cells (DCs) initiate immune responses through their direct interaction with effector cells. However, the mechanism by which DC activity is regulated is not well defined. Previous studies have shown that CTLA4 on T cells regulates DCs function by "cross-talk". We investigated whether there is an intrinsic regulatory mechanism in DCs, with CTLA4 as a candidate regulator.

**Results:**

We confirmed via RT-PCR and flow cytometry the natural expression of CTLA4 on mature DCs derived from human monocytes. Approximately 8% CD1a-positive cells express CTLA4 both on surface and intracellular, whereas 10% CD1a-negative cells express CTLA4 intracellularly, but little expression was observed on the cell surface. The cross-linking of CTLA4 inhibits DCs maturation and antigen presentation in vitro, but does not inhibit endocytosis.

**Conclusions:**

CTLA4 is expressed by DCs and plays an inhibitory role. CTLA4-expressing DCs may represent a group of regulatory DCs. Because of its wide distribution on different cell types, CTLA4 may play a general role in regulating immune responses.

## Background

Dendritic cells (DCs) are sparsely distributed in tissues and the circulation, but they are nevertheless important. They function as professional antigen-presenting cells (APCs) in antigen capture, processing, and presentation to CD4^+ ^and CD8^+ ^T cells [1]. DCs can be produced *in vitro *by a number of procedures, starting from CD34^+ ^hemopoietic progenitor cells (from peripheral blood or bone marrow) cultured with tumor necrosis factor α (TNFα) and granulocyte macrophage-colony-stimulating factor (GM-CSF), or from human blood monocytes cultured with GM-CSF, interleukin 4 (IL-4), or IL-13. Immature DCs (iDCs), which have a high capacity for antigen uptake and processing, but a low capacity to stimulate T-cell proliferation, can be further differentiated *in vitro *to mature DCs (mDCs), which have a high capacity for antigen presentation, by treatment with TNFα, lipopolysaccharide (LPS), IL-1, or CD40L. Many costimulatory factors are expressed by DCs and play important roles in the communication between DCs and immunocompetent cells [2,3]. The functions of DCs are also regulated by the mutual cross-talk between costimulatory molecules [3]. The additional expression of activating costimulatory molecules that favor the interaction between DCs and T cells further enhances the ability of DCs to generate antitumor immune responses. Activating costimulatory molecules that have been upregulated on DCs by genetic engineering include the CD40 ligand (CD40L) [4-6], CD70 [7], 4-1BBL [8], the OX40 ligand (OX40L) [9], and the receptor activator of NF-κB (RANK)/RANK ligand (RANKL) [10].

CTLA4 (CD152) is an inhibitory costimulatory molecule. The expression and function of CTLA4 in T cells have been well studied and the effect of CTLA4 on DCs has also been studied. CTLA4 acts as a ligand to induce interferon γ (IFNγ) production by DCs and to prevent T-cell responses via a mechanism that involves tryptophan catabolism [11]. CTLA4-immunoglobulin (Ig) may inhibit DC function through the B7 receptor on DCs, which indicates cross-talking between costimulatory molecules. A dendritic cell line genetically modified to express CTLA4-Ig suppressed the alloimmune response and prolonged the survival of islet allografts in an allospecific manner [12]. APCs transfected with a gene construct encoding a modified CTLA4 molecule (CTLA4-KDEL) failed to express CD80/86 on their surfaces and were unable to stimulate allogeneic or peptide-specific T-cell responses. The cells also induced antigen-specific anergy of the responding T cells, with no up-regulated expression of the indoleamine 2,3-dioxygenase enzyme [13]. There is evidence that DCs play a central role in immune therapy with CTLA4-Ig insofar as Ko et al. [14] demonstrated that when CD11c^+ ^DCs from collagen-induced arthritis (CIA) mice were treated with CTLA4-Ig and adoptively transferred into mice with CIA, no arthritis developed in association with an increase of the CD4^+^CD25^+^Foxp3^+ ^Treg population. However, in CTLA4-Ig-untreated DC-transferred CIA mice, arthritis developed and then progressed rapidly.

CTLA4 is reported to be expressed on T and B lymphocytes [15,16], monocytes [17], placental fibroblasts [18], human muscle cells [19], CD34^+ ^stem cells, granulocytes [20,21], mouse embryonic stem cells and embryoid bodies [22]. CTLA4 is also expressed in leukemia cells[23] and many cell lines and tumor cells[24]. We hypothesized that CTLA4 is also induced on DCs. The aim of the present study was to investigate the natural presence of CTLA4 on human DCs and the effects of CTLA4 on human DCs differentiation and maturation.

## Results

### Characteristics of DCs

After five days in culture, the cells showed the characteristics typical of iDCs: aggregated cells with extended protrusions visible under microscopy, CD19, CD14, CD3 negative, and CD80, CD86, and HLA-DR-positive phenotype, as demonstrated by FACS (data not shown). After challenge with TNFα plus LPS, the cells were more heterogeneous: some loosely adhered to the plastic surface and others were more buoyant, with long dendrites. The cells were CD83 positive on FACS analysis. CD40, HLA-DR, CD80, and CD86 were strongly expressed (data not shown).

### Cellular expression of CTLA4

**Figure 1:**
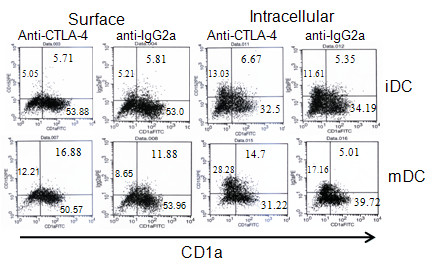
**CTLA4 is naturally expressed by mDCs**. A. Flow-cytometric analysis of DCs expressing CTLA4. iDCs and mDCs were stained on their surfaces or intracellularly with the designated antibodies.

**Figure 2:**
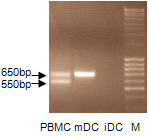
***CTLA4 *mRNA was detected in mature DCs but not immature DCs, as determined by RT-PCR**. The cells were purified by sorting with flow cytometry. iDC: immature DCs; mDC: mature DCs; M: molecular ladder. A representative experiment is shown.

As expected, CTLA4 was expressed intracellularly and on the surfaces of mDCs but not on iDCs, as determined by FACS analysis (Figure 1). A very interesting finding is that both CD1a positive and negative mDCs express CTLA4, but in a different manner. On the surface, about 7.51 ± 1.45% (range 5.1-9.5%) of CD1a-positive mDCs from 10 healthy adults expressed CTLA4 compared to 3 ± 1.38%(1.98-3.9%) CD1a-negative mDCs. Intracellularly, 10.64 ± 2.17% (7.3-13.8%) CD1a-positive mDCS expressed CTLA4 compared to 10.13 ± 3.04%(7.8-13.31%) CD1a-negative mDCs. To verify the specificity of anti-CTLA4 antibody binding, unconjugated CTLA4 antibody was used to block staining with anti-CTLA4-PE antibody. This staining was blocked by the anti-CTLA4 antibody but not by isotype-matched anti-CD3 antibody. *CTLA4 *mRNA was undetectable by RT-PCR in iDCs. mDCs induced by LPS plus TNFα expressed *CTLA4 *mRNA (Figure 2) and this was confirmed by sequencing the PCR products.

### Cross-linking of CTLA4 inhibits DC maturation

**Figure 3:**
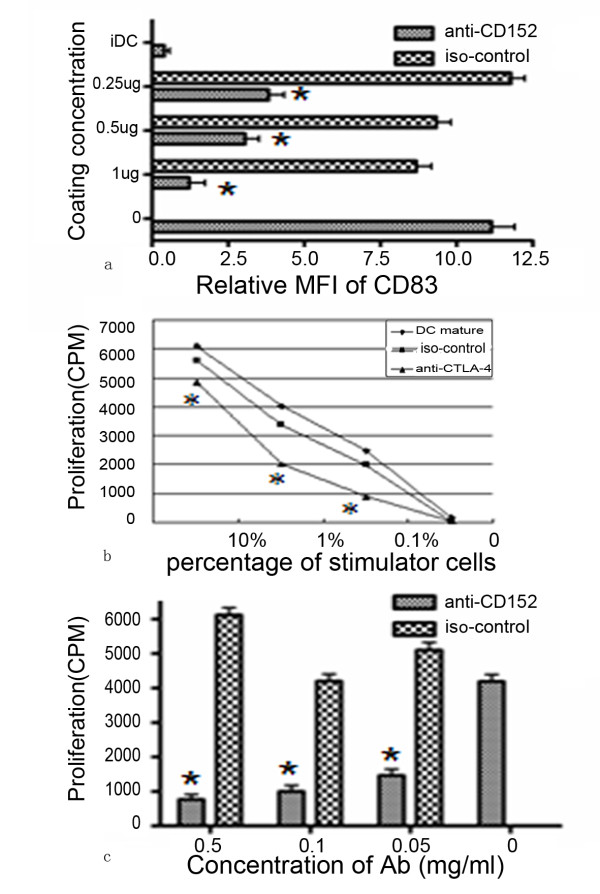
**Cross-linking of CTLA4 inhibits DC maturation (A), the mixed lymphocyte response (B), and antigen presentation (C)**. *: *P *< 0.05 anti-CTLA4 antibody vs control antibody. A representative of three experiments is shown. A: Relative mean fluorescence intensity (MFI; MFI_treated _- MFI_iDC_) of CD83 was measured. DCs were seeded in wells coated with anti-CTLA4 antibody or isotope control antibody at the indicated concentrations, and mature DCs were used as the positive control. B: Mature DCs after challenge in plates coated with anti-CTLA4 or control antibody were used as the stimulators and allogeneic PBMC-derived T lymphocytes as the responders. DCs and T cells were mixed in different ratios with a constant number of T cells (2 × 10^5 ^cells/well). T-cell proliferation was measured in triplicate by the incorporation of [^3^H]-thymidine and is expressed as cpm. C: Effect of CTLA4 cross-linking on the presentation of PPD to specific T-cell lines. After PPD pulsing, DCs were further cultured with maturation-inducing stimuli in plates coated with anti-CTLA4 antibody or control antibody at the indicated concentrations. The cells were then washed extensively and mixed with PPD-specific autologous T-cell lines. Proliferation was assessed as [^3^H]-thymidine uptake.

iDCs were transferred to anti-CTLA4-antibody-coated plates and LPS and TNFα were added. After two days in culture, the cells were harvested for FACS analysis. FACS data revealed that CD83 expression was inhibited in a dose dependent manner by treatment with anti-CTLA4 antibody (Figure 3a). A more than 70% reduction of CD83 means fluorescence intensity (MFI) was achieved after the cells were cultured in plates coated with 1 μg/mL anti-CTLA4 antibody. Because CD83 is commonly used as a marker for mature DCs, the reduced expression of CD83 on DCs suggests the inhibition of DCs maturation. At the same time, CD80, CD86, HLA-DR expression was analyzed. Unexpectedly, there is no significantly effect of anti-CTLA4 antibodies on their expressions. Unlike the CTLA4-KDEL gene transfer which can block CD80 and CD86 expression[13], the crossing-linking of CTLA4 does not alter the basic feature of DC.

### Cross-linking of CTLA4 inhibits MLR and Ag presentation

mDC are potent stimulators of allogeneic T cells. We investigated the possibility that cross-linking of CTLA4 affects the MLR response. Various numbers of stimulators were used and proliferation was determined by [^3^H]-thymidine incorporation. The treated cells showed an impaired capacity to induce MLR (Figure 3b). However, no effect was seen in iDCs treated with anti-CTLA4 antibody (data not shown). Because mDCs are professional APCs, we investigated Ag presentation by mDCs. As CTLA4 is strongly expressed on activated T cells, we treated mDCs separately with either anti-CTLA4 monoclonal antibody or control antibody. As expected, antigen presentation was reduced when mDCs were cultured in anti-CTLA4-antibody-coated plates, which was demonstrated by the reduced proliferative response of specific autologous T-cell lines in the presence of PPD (Figure 3c). The same tendency was observed when tetanus toxoid (50 μg/mL) was used as the challenge (data not shown).

### CTLA4 does not inhibit endocytosis in iDCs

**Figure 4:**
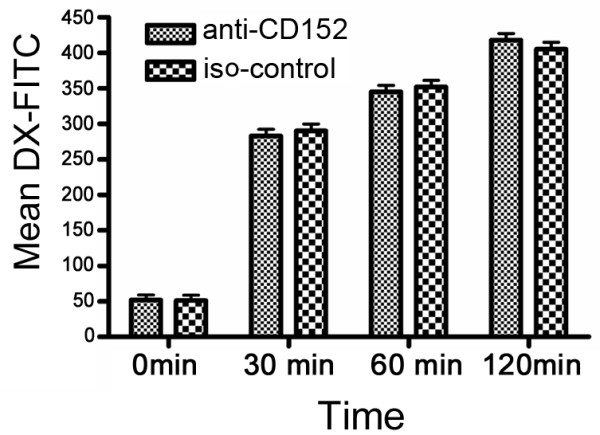
**The culture of immature DCs in wells coated with anti-CTLA4 antibody did not affect their endocytic activity**. Endocytosis was evaluated as the uptake of FITC-DX at the indicated times, which was measured by FACS. The data for one experiment are shown, and are representative of three independent experiments.

The culture of iDCs in anti-CTLA4-antibody-coated plates did not influence their endocytosis (Figure 4). This result is consistent with the expression analysis that showed that CTLA4 was not detectable in iDCs.

## Discussion

Here, we have confirmed that CTLA4 is expressed naturally on mDCs but is undetectable on iDCs. During the revision of this paper, Shankar [25] reported that human PBMC-derived DCs express CTLA4 strongly on their surfaces in response to CH_3_·SAM (self-assembled monolayers) contact (a kind of biomaterial) when compared with all other treatments. This result is consistent with our data.

CD1a was widely used as human DCs marker several years ago. In recent years, CD1a negative DCs have been identified[26,27]. It is clear now that DCs are a heterogeneous population with peculiar phenotypic and functional features, regardless of whether they are generated from human monocytes or CD34^+^-hematopoietic progenitors[28]. However, there is no full picture of DCs subgroups to date. Surface expression of CTLA4 was favored on CD1a-positive mDCs whereas intracellular expression occurred not only in CD1a-positive, but also in CD1a-negative mDCs. The percentage of CTLA4-positive staining was slightly higher for intracellular than for surface staining of CD1a-positive cells. As intracellular staining covers both the surface and the cytoplasm, surface positive mDCs may represent a group of regulatory mDCs, which may be the same as the situation for T cells. About 10% CD1a-negative mDCs expressed CTLA4 intracellularly, but not on the cell surface. This group of cells may represent a particular population with unknown function. Further studies to determine if there are regulatory DCs would be very interesting. It is also important to define the characters of CTLA4-positive CD1a positive or negative mDCs via phenotype and function analyses.

The precise function of CTLA4 in non-T cells is largely unknown. It is reasonable to infer that CTLA4 plays an inhibitory role via different mechanisms in different cell types, but some mechanisms may also be shared by different cell types. According to the limited data available, CTLA4 plays a negative role in the cell activity of non-T cells. A balance between stimulatory and inhibitory signals is required for effective immune responses and the B7 and CD28 superfamilies are major regulators of this critical balance, because key positive and negative second signals are provided by these pathways. The function of CTLA4 in T cells has recently been reviewed [29] and summarized as seven activities: 1. increases the threshold for cytokine production and proliferation; 2. inhibits cell-cycle progression and transcription factors NF-κB, NF-AT, and AP-1; 3. induces indoleamine 2,3-dioxygenase release by dendritic cells; 4. modulates the composition and cell-surface expression of lipid rafts; 5. upregulates LFA1-mediated adhesion via the activation of RAP1; 6. increases cell motility and reverses the T-cell-receptor-induced stop signal; and 7. reduces T-cell-APC contact time. Moreover. CTLA4 affects T cells motility [30,31]. Another function of CTLA4 is in the protection of cells from apoptosis. Hoff et al. demonstrated that the protection of cells from apoptosis by CTLA4 is dependent on the suppression of the FAS/FASL system and is mediated by the PI3K-dependent activation of the kinase AKT, leading to the inhibition of the proapoptotic molecule BAD [32]. All these functions may be used as references in determining the CTLA4 function in DCs. Although the present data and other studies[12] suggest CTLA4 plays an inhibitory role in the main functions of DCs, the mechanisms involved remain to be identified, especially the signal pathway in DCs.

Two types of bidirectional cross-talk between different cells play important role: 1. reverse signaling such as CD47 and signal-regulatory protein-a (SIRP-a) [33], CD40 and CD154 [34], and CD200 and CD200R [35]. 2. coexpression of some ligands and their receptors on the same cells. We have observed that T cells express not only CD28 and CTLA4, but also CD80 and CD86 when activated [36]. It has been reported that activated B cells not only express CD80 and CD86, but also CTLA4 [15]. CTLA4, acting as ligand, could regulate DC function [11-14]. The finding of natural expression of CTLA4 by DCs supports bidirectional communication as a possible mechanism. The bidirectional signaling mediated by pairs of membrane-anchored coreceptor molecules is likely to be an important feature of the DC-T-cell interaction.

DCs are powerful APCs, and can present antigens to CD4^+ ^and CD8^+ ^T cells while delivering the costimulatory signals necessary for T-cell activation. CTLA4 expressed on DCs could finely regulate the functions of DCs. The results of our experiments, in which we used an anti-CTLA4 antibody to cross-link the surface CTLA4 molecules on DCs, suggest that the function of CTLA4 on DCs is to down-regulate their maturation and antigen presentation. The results of our functional studies are also supported by the transfection of a gene construct [13] that encodes a modified CTLA4 molecule (CTLA4-KDEL). Although less than 10% of DCs expressed CTLA4, the power of CTLA4 is still great. Beside direct contact, the mechanism for this potent inhibitory role may involve cytokine secretion by CTLA4-expressing DCs. We propose that CTLA4-expressing DCs may represent a group of regulatory DCs that can regulate DCs function. Future work will involve characterization of phenotypes and functions of CTLA4-expressing DCs.

Conclusions: DCs are specialized regulators of innate and adaptive immunity. Because CTLA4 is expressed not only on T cells but also on various other cell types, we believe that CTLA4 plays a global role in the finely tuned regulation of cell activity in vivo. CTLA4-expressing DCs may represent a group of regulatory DCs. Our demonstration of the natural, functional expression of CTLA4 on DCs increases the evidence of general role of CTLA4 and further confirms the importance of CTLA4 in the immune system.

## Methods

### Cytokines and reagents

Recombinant human GM-CSF (1.1 × 10^4 ^U/μg), recombinant human IL2 (2 × 10^6 ^U/mg), and recombinant human TNFα (specific activity >2 × 10^7 ^U/mg) were obtained from PeproTech (London, UK). Recombinant human IL4 was obtained from R&D Systems (R&D Systems, Minneapolis, MN). LPS was from *Escherichia coli *(055:B5; Sigma, St Louis, MO). *Mycobacterium tuberculosis *purified protein derivative (PPD), manufactured at the Statens Serum Institute, Denmark, was supplied by SBL Vaccin AB, Stockholm, Sweden. Purified anti-human CTLA4 (CTLA4) and mouse IgG2a κ isotope controls or anti-human CD3(IgG2a) were purchased from Pharmingen (San Diego, CA, USA).

### DCs culture

Highly enriched monocytes (>80% CD14^+^) were obtained from buffy coats on Ficoll gradients and purified by peripheral blood mononuclear cell (PBMC) adherence. Nonadherent cells were depleted by washing three times with phosphate-buffered saline (PBS). The adherent cells were recovered; CD2^+ ^and CD19^+ ^cells were depleted with a negative selection kit (Dynabeads M450; Dynal, Oslo, Norway). This procedure resulted in a 97% pure CD14^+ ^monocytes preparation. Monocytes were cultured for seven days at 1 × 10^6^/mL in 24-well multi-well tissue-culture plates (Falcon, Becton Dickinson, Stockholm, Sweden) in RPMI 1640 medium (Invitrogen AB, Stockholm, Sweden,) in 10% fetal calf serum (FCS) supplemented with 60 ng/mL GM-CSF and 50 ng/mL IL4. The medium was changed every second day by removing half the medium and adding freshly made medium supplemented with full concentrations of cytokines. To obtain mDCs, 100 ng/mL LPS and 10 ng/mL TNFα were added.

### RT-PCR amplification and sequencing of human *CTLA4*

The CD1a-positive cells were sorted on a MoFlo high-speed cell sorter (Dako, Glostrup, Denmark) equipped with a Coherent Innova 70C laser tuned to 488 nm. Fluorescein isothiocyanate (FITC) fluorescence emission was detected with a 530/40 optical filter. Rhodamine phycoerythrin (RPE) fluorescence emission was detected with a 580/30 optical filter. Mature DCs were sorted based on gates set on forward scatter versus side scatter and CD83-PE expression. iDCs were sorted based on gates set on forward versus side scatter and CD1a FITC expression. Doublets were discriminated and excluded using a gate set on forward scatter versus pulse width. No T cells were identified by fluorescence-activated cell sorting (FACS) analysis. Purified DCs (2 × 10^6^) were lysed in 500 μL of a total RNA isolation reagent (Ultraspec-II RNA, Biotech, Stockholm, Sweden). cDNA was prepared with the RNA PCR Core kit (PerkinElmer, Foster City, CA). The primers used to amplify the entire coding sequence of *CTLA4 *were 5'-ATGGGCCACACACGGAGGCA-3' and 5'-TACACTTTCCCTTCTCAATCTCTCAT-3'. The PCR conditions were as follows: 94°C for 5 min, 35 cycles of 94°C for 1 min, 56°C for 1 min, and 72°C for 1.5 min, followed by a final extension at 72°C for 5 min. The amplified fragments were separated and identified on a 2% GTG agarose gel. The fragments were subcloned into a TA cloning vector (PCRII, Invitrogen, Leek, Netherlands). Sequencing was performed with the ABI PRISM BigDye Terminator Cycle Ready Reaction Kit (PerkinElmer, Wellesley, MA).

### FACS analysis

Cell staining was performed with CD1a-FITC (clone WM35 from Nordic BioSite AB), CD3-PerCP, CD14-FITC, CD19-FITC, CD83-PE (Clone HB15), CD40-PE, CD80-FITC, CD86-FITC, CD86-PE, CD14-PE, HLA-DR-FITC, HLA-DR-PerCP, CD152-PE (BD Biosciences, Franklin Lakes, NJ). Intracellular staining of CTLA4 was as described previously [37]. The results are expressed as the percentages of positive cells or as mean fluorescence intensity (MFI), calculated according to the formula: MFI = mean fluorescence (sample) - mean fluorescence (control).

### Cross-linking of CTLA4

DCs were collected, counted, and incubated in anti-CTLA4-antibody-coated 24-well plates in complete medium (1 × 10^6 ^cells/well) at 37°C. As controls, DCs were incubated with an equal amount of coated-anti-mouse-IgG2a antibody or left untreated.

### Inhibition of DCs maturation

TNFα (10 ng/mL) and LPS (100 ng/mL) were added to induce the maturation of the DCs in the wells coated with different concentrations of anti-CTLA4 or control antibody (anti-mouse-IgG2a). The cells were recovered after 48-72 h of culture. CD83 expression was used as the marker of maturity and was detected by FACs.

### Endocytosis

Mannose-receptor-mediated endocytosis was measured as the cellular uptake of FITC-Abtran (FITC-DX; M_*r *_40,000; Sigma) and quantified by flow cytometry. Approximately 2 × 10^5 ^iDCs pretreated with anti-CTLA4 antibody or the control antibody were incubated in medium containing FITC-DX (1 mg/mL) for 0, 60, or 120 min. After incubation, the cells were washed twice with PBS to remove excess Abtran and fixed in cold 1% formalin. The quantitative uptake of FITC-DX by the cells was determined with FACS. At least 10,000 cells were analyzed per sample.

### Mixed lymphocyte reaction (MLR)

DCs were cultured in GM-CSF plus IL4 for seven days, after which maturation factors (10 ng/mL TNFα and 100 ng/mL LPS) were added for two days. The mDCs were challenged (or stimulated) with 2 μg/mL coated-anti-CTLA4 antibody or control antibody (anti-mouse-IgG2a) for 24 h. The DCs were collected, irradiated (25 Gy), washed extensively, and added in graded doses to 2 × 10^5 ^responder cells in 96-well flat-bottomed micro test plates (Costar, Cambridge, MA) in complete medium containing 5% autologous plasma instead of 10% FCS. The responder cells were purified allogeneic T cells that were depleted of APCs by passage with CD14- and CD19-coated Dynabeads. Each group was treated in triplicate. Thymidine incorporation was measured on day 5 with a 16 h pulse of [^3^H]-thymidine (1 μCi/well, specific activity 5 Ci/mM; Amersham Life Science, Buckingham, UK).

### Antigen (Ag) presentation assay

PPD-responsive T-cell lines were generated in our laboratory by culturing mononuclear cells with PPD (50 μg/mL) for one month in the presence of IL2 (100 U/mL). The medium was changed twice a week for medium with replenished cytokines. Peripheral monocytes for DCs were obtained from the same donor as T cells. After seven days, the DCs were preincubated with PPD (50 μg/mL) for 12 h in serum-free RPMI 1640 medium to take up the antigen. Then medium was replaced with medium supplemented with maturation-inducing stimuli (10 ng/mL TNFα and 100 ng/mL LPS) and the cells were cultured in the presence or absence of coated-anti-CTLA4 antibody for 48 h. Isotope control(anti-CD3) antibody was used as the antibody control. The DCs were then washed extensively and cocultured with a PPD-responsive autologous T-cell line for 72 h in 96-well microtiter plates. Their [^3^H]-thymidine uptake was measured during the last 12 h of culture.

### Statistical analysis

Results were expressed as the mean ± SD. Variance analysis was used for statistical comparisons between groups by Student's t-test. Statistical significance was defined as a *P *value of < 0.05.

## Authors' contributions

XW conceived of the study, participated in its design and coordination and wrote the manuscript. ZF was involved in drafting the manuscript. DA carried out the cellular studies. AV carried out the cell sorting. ZN carried out the molecular genetic studies. AL participated in the design of the study. All authors read and approved the final manuscript.
